# Editorial: The interconnectedness of personality and language

**DOI:** 10.3389/fpsyg.2023.1215174

**Published:** 2023-07-11

**Authors:** Petar Čolović, Boele De Raad, Alexandre José De Souza Peres, Dušica Filipović Đurđević, Vesna Lazović, Boris Mlačić

**Affiliations:** ^1^Department of Psychology, Faculty of Philosophy, University of Novi Sad, Novi Sad, Serbia; ^2^Department of Psychology, University of Groningen, Groningen, Netherlands; ^3^Federal University of Mato Grosso do Sul, Campo Grande, Brazil; ^4^Faculty of Philosophy, University of Belgrade, Belgrade, Serbia; ^5^Department of English, Faculty of Arts, University of Ljubljana, Ljubljana, Slovenia; ^6^Institute of Social Sciences Ivo Pilar (IPI), Zagreb, Croatia

**Keywords:** personality, language, natural language processing, psycho-lexical, cross-cultural

Our principal idea behind this special issue was to highlight the importance of connecting the classic psycho-lexical approaches in personality psychology with emerging tendencies in natural language processing. Hence our “target contributing field” was initially planned as multi-disciplinary, hoping that psychologists, linguists, and researchers from bordering fields would help us picture a map of the area more clearly, and to understand the potential and obstacles for field development. To provide a more compelling rationale and hopefully a clearer view of the topics in this Frontiers issue, we opted for a bibliometric approach, more specifically thematic mapping. For additional information on the procedure, we point the readers to the materials available on our OSF project page (https://osf.io/zvkag/).

Nine papers are published in this issue, with contributions from 41 co-authors. The articles are authored by three to nine co-authors per paper, with one single-authored article. Authors' affiliations comprise sixteen research institutions from Australia, Croatia, the Czech Republic, Germany, Hungary, Korea, the Netherlands, New Zealand, Poland, Serbia, and the United States of America. The proportion of international authorship is 33%, suggesting that one-third of the papers are produced in international collaborations. Six articles are original research papers, one an opinion article, one a method paper, and one a brief report. This overview suggests that this Frontiers issue may be considered as an international effort, offering diverse perspectives on the issues connecting personality and language. Our editorial team is especially honored by attracting the attention of esteemed authors in linguistics, psychology, and information science, making this effort even more valuable.

While it is a subtle and delicate task to summarize the current contributions conceptually, we take the liberty to make a brief comment on the concepts covered in this issue, as we see it presently. Two papers are firmly rooted in the long-standing tradition of lexical research in personality, though both seem to cross, challenge, or redefine the current research practices in the field. A paper by Volungevičiene et al. explores the social effects (a seldomly investigated descriptor category) in a specific language and cultural context. An opinion piece (Saucier) by one of the leading experts in the field is an overview of the results and methodological practices in the psycho-lexical tradition, and simultaneously an outlook into its future. The psycho-lexical tradition is, through its methods and concepts, present in the paper by Fischer et al. however, this paper extends the values-related research to autobiographical narratives. Similarly, Brauer et al. explore the concept of playfulness in natural language, using a reliable but perhaps so far sparsely used automated language analysis paradigm; this is a prime example of using NLP procedures to gain advanced knowledge of a concept. Spitzley et al. use the same procedure (LIWC) along with a SPLICE measurement technique to explore the diversity of language styles and their relations to the Big Five personality traits. The use of state-of-the-art analytic procedures is revisited in a paper by Dai et al. relating natural language use in a specific setting with the HEXACO model of personality. Genkova et al. focus on language and culture as landmarks of a multi-cultural and multi-language area such as Eastern Europe, validating the constructs developed in Western culture, in a current and valuable etic- study. Calić et al. venture further into language-related communication, and beyond it, by exploring the paralinguistic cues in a sample of adults with moderate intellectual disabilities. Jang et al. propose a study protocol on the relations of the Five-Factor Model of personality traits (assessed through NLP) and psychological distress.

Consistent with the authorship, the topical structure of our thematic issue is diverse. Opting to describe it using bibliometrics again, we chose the bibliographic coupling technique (Van Eck and Waltman, 2014, 2017; Aria and Cuccurullo, 2017; Moral-Muñoz et al., 2020), whereby the documents' similarity was estimated using the degree of their references overlap Three thematic clusters, defined by authors' keywords, emerged in our collection. The first one is marked broadly as “language and personality”; the other two are “natural language processing” and “personality prediction”. While we believe that they conveniently account for the wide range of our topics, what particularly interested us is how these topical patterns correspond with the conceptual structure of the field, as the field and its structure were before our first call for contributions was dispatched.

The thematic mapping procedure enables the (tentative) topic allocation into one of four quadrants based on the combined scores on cluster density (standing for topic development) and cluster centrality (standing for topic relevance). The quadrants are labeled as “niche themes” (high density, low relevance), “motor themes” (both scores high), “emerging or declining themes” (both scores low), and “basic themes” (low density, high relevance).

The 2021 map of the personality-language field suggests well-populated “niche” and “basic” categories, with a multi-topic (but mostly NLP and machine learning-oriented) cluster almost at the center. Although the motor themes quadrant is scarcely populated, two clusters are bordering on it: a predominantly NLP-themed one and a second, containing terms typical of individual differences and foreign language learning.

The projection of our 2023 themes to the 2021 conceptual map, shows that the broad “language–personality” cluster is located between the “niche” and “motor” quadrants. We believe that such a result may be in favor of the emerging relevance of this stream of research; we believe that the studies that are underway will be able to help increase it. Natural language processing is embedded within the “basic” topics, somewhat unexpectedly surrounded by “core” personality psychology concepts. This may suggest that a methodological shift toward advanced NLP techniques has to be reckoned with in personality studies. Personality prediction is located between the niche and emerging themes, suggesting that the topic “density,” a.k.a. the number of studies within the research line, would be welcome, in order to define the personality prediction conceptual status better.

Personality and language, as meta-concepts, have complex and nuanced meanings that complement and inform one another. These papers comprise a diversity of topics, concepts, and research methods, all related to personality constructs but striving to push forward the boundaries of understanding personality through language and vice versa. Such a diversity points to the interest and enthusiasm of the authors in multidisciplinary settings to continue exploring these and related concepts. We hope that this Frontiers issue has encouraged the researchers to delve into this territory further. If we have managed to accomplish that task, our humble contribution to the field has already been made.

## Author contributions

PČ wrote the first draft of the manuscript and performed the analyses. DF and PČ managed the supplementary material. BD, VL, AP, and BM modified the draft version and articulated the final version. All authors contributed to the article and approved the submitted version.
